# Editorial: Multitasking Biomolecules in Human Pathologies: Known Players on Their Unexpected Journeys

**DOI:** 10.3389/fmed.2020.00478

**Published:** 2020-08-21

**Authors:** Malgorzata Wygrecka, Djuro Kosanovic, Grazyna Kwapiszewska, Klaus T. Preissner

**Affiliations:** ^1^Department of Biochemistry, Universities of Giessen and Marburg Lung Center, Giessen, Germany; ^2^Department of Pulmonology, I. M. Sechenov First Moscow State Medical University (Sechenov University), Moscow, Russia; ^3^Ludwig Boltzmann Institute for Lung Vascular Research, Graz, Austria

**Keywords:** moonlighting, multitasking, drug design, drug development, human diseases

Over the last few years a number of studies have brought forward new and exciting discoveries in biomolecule research and have changed the old paradigm “one molecule, one function, one trait.” Today, we know that exactly the same biomolecule may operate at different locations, utilize different substrates or binding factors, and thus perform multiple totally unrelated functions. Interestingly, these properties are not only reserved for proteins, but also DNA and RNA molecules as well as diverse classes of metabolites possess the ability to multitask. The discovery of multifunctionality of biomolecules, as well as their “moonlighting business,” led to the identification of new interaction networks and provided new insights into the pathogenesis of many disorders. In addition, it opened new perspectives for the diagnosis and treatment of the diseases and thus for drug repurposing and development (1, 2). In the Research Topic of the *Frontiers in Medicine* entitled “Multitasking Biomolecules in Human Pathologies: Known Players on Their Unexpected Journeys” new faces of diverse molecules are presented and discussed in the context of their significance for human pathologies.

Circular RNAs (circRNAs) are non-coding RNAs with a closed-loop structure generated from pre-mRNAs by backsplicing. Although circRNAs are mainly known as miRNA sponges, recent findings provide solid evidence for their multitasking nature. CircRNAs regulate fundamental cellular processes such as transcription, splicing, mRNA turnover, translation and posttranslational signaling. Thus, it is not surprising that circRNAs are involved in the development of numerous human pathologies, including cardiovascular diseases, diabetes, or cancer (3). Wang et al. add acute pancreatitis (AP) to the list of the diseases associated with the alterations in circRNA levels. The authors not only describe high levels of circRNA circHIPK3 in serum of patients suffering from AP, but also provide mechanistic insights into the role of circHIPK3 in this pathological condition. They report that circHIPK3 inhibits expression of miR193a-5p in acinar cells thus enhancing the release of inflammatory mediators consequently leading to gasdermin D-driven pyroptosis. This study thus supports the evidence that the alterations in the expression of circRNAs may associate with human pathologies and encourages further efforts on the development of therapies to target these molecules.

Multifunctionality, however, is not only reserved for RNA molecules, but it is also a feature of numerous intra- and extracellular proteins. Enzymes, transcription factors, translation elongation factors, or structural proteins were identified to perform diverse unrelated functions. In this regard, Didiasova et al. highlights the multitasking activities of the glycolytic enzyme enolase. These authors couple specific, unrelated functions of enolase to its different sites of action inside or outside cells, consequently bringing forward the concept of “molecular moonlighting” for this protein. Molecular moonlighting refers to the practice of holding a second night-time job, in addition to a regular day-time job. In contrast to multitasking, which describes the ability to perform more than one task at the same time, moonlighting accentuates spatial separation of diverse activities (4). Following this definition, Didiasova et al. emphasize that in mitochondria enolase stabilizes the organelle's membrane, in cytoplasm contributes to glycolysis, regulates cytoskeletal dynamics and protects against stress conditions, while on the extracellular side of the cell membrane modulates pericellular proteolysis and in the extracellular milieu controls inflammation.

The spatial separation of the activities conducted by moonlighting molecules is also highlighted by Kapurniotu et al. who describe multiple faces of high-mobility group box protein-1 (HMGB-1) and macrophage migration-inhibitory factor (MIF), thereby taking the reader into the intriguing world of danger-sensing molecules, known as alarmins. Alarmins have a physiological role inside cells, however, once released into the environment, they acquire the ability to moonlight and fulfill other, unrelated jobs. HMGB-1 and MIF amplify inflammatory responses outside cells, but inside cells, HMGB-1 regulates transcription and MIF controls cell survival by binding to CSN5, superoxide dismutase or even DNA. Dependency between localization and function has been also proposed for translation elongation factors, thus enlarging the repertoire of their functions to the involvement in RNA splicing, microRNA turnover, or DNA damage response (5).

High multifunctionality of enolase, HMGB-1, and MIF makes them attractive therapeutic targets. Indeed, numerous preclinical studies documented translational potential of strategies aiming at blocking enolase, HMGB-1, and MIF (6–8). However, multifunctionality of biomolecules has also numerous drawbacks and it must be taken with caution in regard to drug-design and drug-optimization. As moonlighting molecules play key roles in many physiological processes, specific pharmacological interventions tackling them need to discriminate between their “good” and “bad” function, and this still remains a challenge for drug development. The “bad” side of moonlighting proteins is highlighted by Lam and Roudier who shed light on cardiovascular side-effects of anti-cancer therapies targeting the E3 ubiqutin ligase murine double minute 2 (MDM2). MDM2 regulates key cellular process, including DNA repair and synthesis, transcription, hypoxia signaling, and apoptosis, in a p53-dependent as well as a p53-independent manner. The aforementioned spectrum of activities makes MDM2 an attractive target for anti-cancer therapies, however, recent studies raise awareness about cancer-unrelated activities of MDM2. In fact, MDM2 seems to be a part of the “social” network in cardiovascular homeostasis during the body's lifespan. This aspect forces the rethinking of current therapeutic strategies targeting MDM2 in cancer.

The Research Topic collection is closed by an exciting review article by Espinosa-Cantu et al. who discuss moonlighting in the context of pleiotropy, multidomain structure of proteins, and promiscuity thereby questioning a clear-cut definition of molecular multifunctionality. These authors also highlight how moonlighting and multitasking proteins evolved and how they currently influence systems biology approaches, large-scale genomic, transcriptomic and proteomic studies, the design of new proteins, or the development of new therapeutics for the use in diverse human pathologies.

The discovery of moonlighting biomolecules has opened new horizons for the diagnosis and treatment of diseases including those, wherein interdisciplinary treatment approaches are warranted. Further studies addressing the molecular mechanisms responsible for switching between different moonlighting functions of biomolecules will aid research in medicine and open new exciting avenues for the treatment of multitude of human disorders.

## Author Contributions

All authors listed have made a substantial, direct and intellectual contribution to the work, and approved it for publication.

## Conflict of Interest

The authors declare that the research was conducted in the absence of any commercial or financial relationships that could be construed as a potential conflict of interest.
